# Inducible Bronchus-Associated Lymphoid Tissue Elicited by a Protein Cage Nanoparticle Enhances Protection in Mice against Diverse Respiratory Viruses

**DOI:** 10.1371/journal.pone.0007142

**Published:** 2009-09-23

**Authors:** James A. Wiley, Laura E. Richert, Steve D. Swain, Ann Harmsen, Dale L. Barnard, Troy D. Randall, Mark Jutila, Trevor Douglas, Chris Broomell, Mark Young, Allen Harmsen

**Affiliations:** 1 Department of Veterinary Molecular Biology, Montana State University, Bozeman, Montana, United States of America; 2 Center for Bio-Inspired Nanomaterials, Montana State University, Bozeman, Montana, United States of America; 3 Department of Chemistry and Biochemistry, Montana State University, Bozeman, Montana, United States of America; 4 Department of Plant Sciences, Montana State University, Bozeman, Montana, United States of America; 5 Institute for Antiviral Research, Utah State University, Logan, Utah, United States of America; 6 Division of Allergy, Immunology and Rheumatology, University of Rochester Medical Center, Rochester, New York, United States of America; Centre de Recherche Public de la Santé (CRP-Santé), Luxembourg

## Abstract

**Background:**

Destruction of the architectural and subsequently the functional integrity of the lung following pulmonary viral infections is attributable to both the extent of pathogen replication and to the host-generated inflammation associated with the recruitment of immune responses. The presence of antigenically disparate pulmonary viruses and the emergence of novel viruses assures the recurrence of lung damage with infection and resolution of each primary viral infection. Thus, there is a need to develop safe broad spectrum immunoprophylactic strategies capable of enhancing protective immune responses in the lung but which limits immune-mediated lung damage. The immunoprophylactic strategy described here utilizes a protein cage nanoparticle (PCN) to significantly accelerate clearance of diverse respiratory viruses after primary infection and also results in a host immune response that causes less lung damage.

**Methodology/Principal Findings:**

Mice pre-treated with PCN, independent of any specific viral antigens, were protected against both sub-lethal and lethal doses of two different influenza viruses, a mouse-adapted SARS-coronavirus, or mouse pneumovirus. Treatment with PCN significantly increased survival and was marked by enhanced viral clearance, accelerated induction of viral-specific antibody production, and significant decreases in morbidity and lung damage. The enhanced protection appears to be dependent upon the prior development of inducible bronchus-associated lymphoid tissue (iBALT) in the lung in response to the PCN treatment and to be mediated through CD4+ T cell and B cell dependent mechanisms.

**Conclusions/Significance:**

The immunoprophylactic strategy described utilizes an infection-independent induction of naturally occurring iBALT prior to infection by a pulmonary viral pathogen. This strategy non-specifically enhances primary immunity to respiratory viruses and is not restricted by the antigen specificities inherent in typical vaccination strategies. PCN treatment is asymptomatic in its application and importantly, ameliorates the damaging inflammation normally associated with the recruitment of immune responses into the lung.

## Introduction

There is currently no immunoprophylactic strategy to generate broad spectrum protection against diverse respiratory viral pathogens. Conventional strategies to protection against respiratory pathogens have relied on vaccination against individual virus strains or the use of drug based therapies. However, the efficacy of these immunoprophylactic strategies is often significantly limited by either difficulty in engineering effective vaccines to some respiratory viruses such as respiratory syncytial virus, or because of evasion of acquired immunity as a result of antigenic variation of circulating respiratory viral pathogens (H1N1 swine influenza pandemic, 2009). In addition, conventional strategies are generally not applicable to newly emerging viruses (SARS outbreak, 2003) since their sudden appearance does not allow for time to develop a vaccine.

Since many pathogens either directly infect the mucosal epithelium or enter a host by penetration of mucosal surfaces, the induction of effective pathogen-specific immunity at local mucosal sites is becoming a desirable objective of novel vaccination strategies. Mucosal, as opposed to systemic vaccination is often more efficacious against mucosal pathogens due to their ability to induce secretory IgA [1], [2]. Despite such an advantage, vaccination of mucosal epithelial surfaces, especially those of the lung, pose significant safety concerns due to the fragility of the tissue and the likelihood of triggering unwanted pulmonary inflammation [3]. Consequently, it is critical that the development of novel methods for pulmonary immunization focus not only on the generation of effective mucosal immunity but also on the amelioration of concomitant inflammatory responses. Thus, new immunoprophylactic strategies that facilitate the local generation of protective immunity against a broad spectrum of lung pathogens and are able to diminish any detrimental inflammatory sequelae associated with the onset of primary adaptive and/or innate immune responses at the site of infection would be highly advantageous.

Therapies that cause the formation of inducible bronchus-associated lymphoid tissue (iBALT) may provide an alternative immunoprophylactic approach for broad spectrum viral protection in the lung. Although iBALT development is known to occur in association with pulmonary immune responses [4], [5], the significance of the role of iBALT in the resolution of infections has only recently been investigated, most notably in its role in adaptive immunity to influenza virus infections [6], [7]. Remarkably, iBALT can replace the function of the spleen and other secondary lymphoid tissues in primary adaptive immune responses [7] and also support the maintenance and re-expansion of adaptive immune memory responses following influenza virus infections [6]. The association of iBALT with lung pathologies in autoimmune diseases [5], or the necessity of infection for its induction [4], has diverted attention from the immunoprophylactic potential of iBALT. Here, we demonstrate that iBALT can be induced asymptomatically without infection or ensuing pathology, and that pre-existing iBALT enhances and accelerates the primary adaptive and likely innate immune responses to respiratory viruses in the naïve host.

## Results

### Protection against viral or bacterial infection

**Figure 1:**
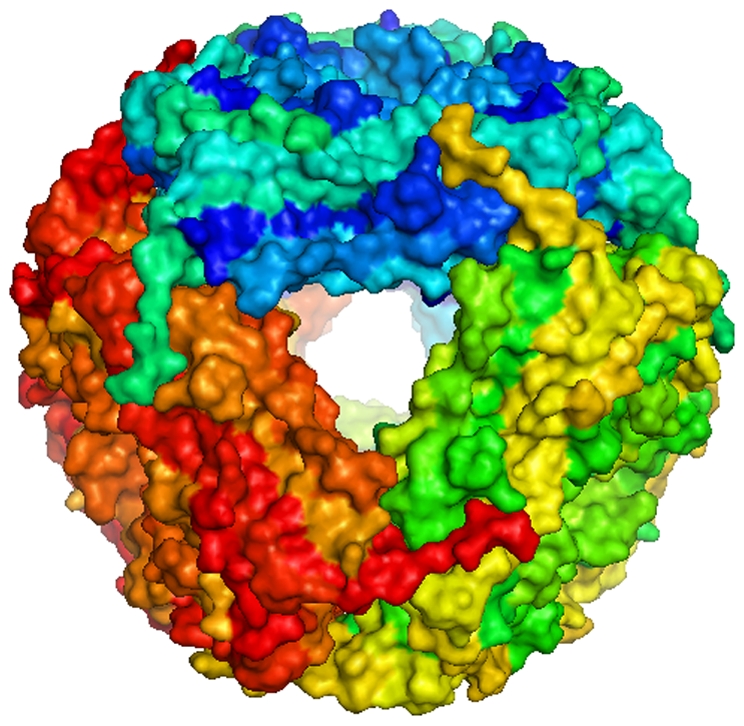
Representative diagram of assembled protein cage nanoparticle showing individual protein subunits.

**Figure 2:**
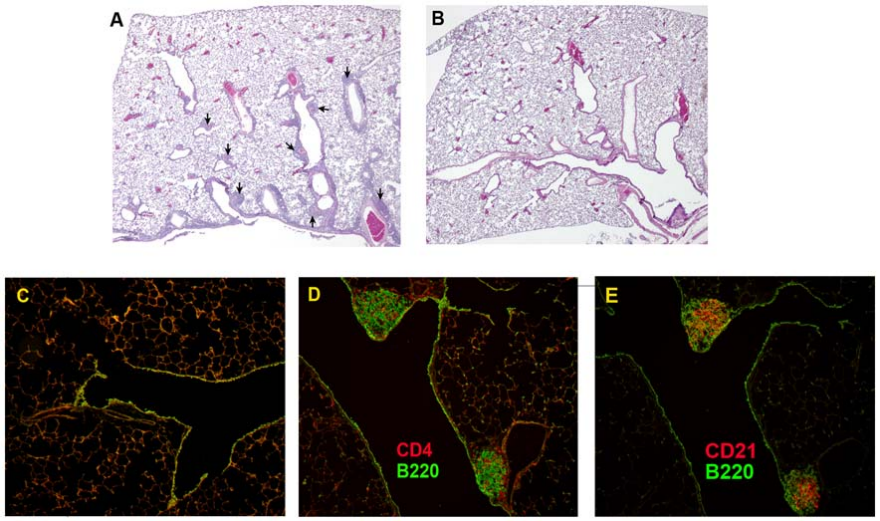
Induction of pulmonary iBALT structures in response to treatment with PCN. iBALT structures (arrows) form in association with airways and blood vessels of the lung in response to 5 PCN treatments (A), whereas no iBALT structures were induced in response to 5 PBS treatments (B and C). PCN-induced iBALT structures contained CD4+ T cells, B220+ B cells (D), and CD21+ follicular dendritic cells (E).

The protein cage nanoparticle (PCN) utilized here was derived from the small heat-shock protein (sHsp 16.5) of the hyperthermophilic archaeon *Methanococcus jannaschii* and is unrelated to other classes of heat-shock proteins (Hsp 50, 60, 70) [8]. This non-infectious PCN is comprised of 24 identical subunits that spontaneously self-assemble into hollow, spherical protein cages that are 12 nm in diameter (Figure 1). Interestingly, pulmonary instillation of the PCN induced iBALT development within the lung parenchyma (Figure 2A) whereas treatment with PBS vehicle control did not induce any detectable iBALT formation (Figure 2B and 2C). PCN-induced iBALT structures formed in the submucosa adjacent to blood vessels and lymph ducts associated with bifurcations of the conducting airways down to the level of the terminal bronchioles. The PCN-induced iBALT structures contained B cells, CD4 T cells, follicular dendritic cells (Figure 2D and 2E), and CD8 T cells (data not shown), similar to that of iBALT structures induced by influenza infection [7] and seen in other non-inducible secondary lymphoid tissues [9], [10].

**Figure 3:**
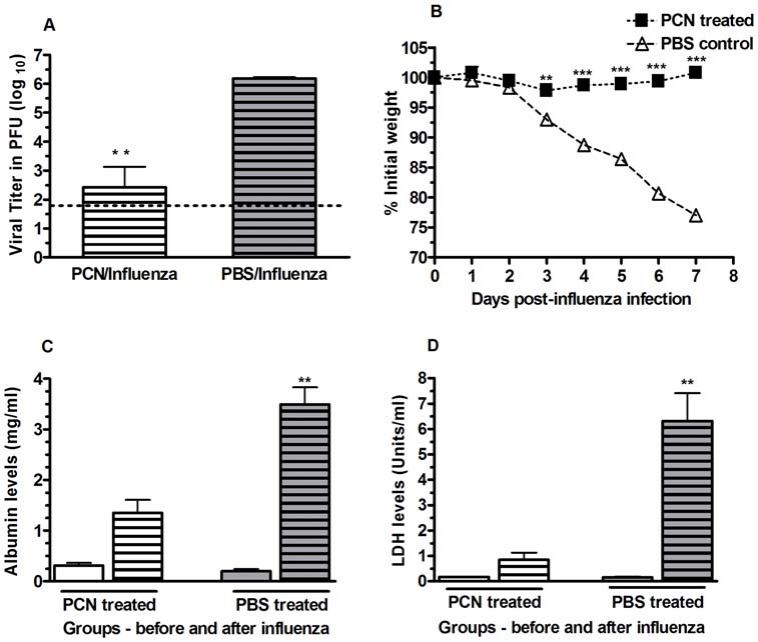
Resolution of PR8 influenza virus infection is enhanced in mice treated with PCN prior to infection. A) Viral recovery from the lungs of mice treated with PCN 9x prior to infection is significantly enhanced; **, P = 0.002; dotted line is limit of assay detection. B) PCN-treated mice did not lose body weight during resolution of subsequent influenza virus infection; **, P = 0.0024; ***, P<0.0001. No lung damage in association with the PCN treatment is evident, as indicated by the equivalent serum albumin (C) and lactate dehydrogenase (D) levels observed in the in the BALF of PCN treated mice (clear bars) and PBS treated mice (shaded bars) prior to infection. Following infection, serum albumin and LDH levels in the recovered BALF of PCN treated mice (clear bars with lines) is significantly less than that detected in the PBS treated mice (shaded bar with lines); **, P = 0.003.

Mice treated with PCN were protected against a spectrum of respiratory viral pathogens. In mice treated 9x with the PCN, the protection against a subsequent primary influenza virus infection was characterized by a significantly greater reduction in viral burdens by day 7 post-infection (Figure 3A), and by the absence of any weight loss (Figure 3B), and lung damage, as indicated by the decreased levels of albumin (Figure 3C) and lactate dehydrogenase (LDH) (Figure 3D) recovered from the broncho-alveolar lavage fluid (BALF) [11], [12]. Although we also observed an enhancement of protection when 100 ug of the PCN was administered 3x prior to influenza infection, we subsequently found that 5 doses of 100 ug/dose of the PCN was optimal to achieve the accelerated viral clearance and the reduced morbidity. In addition, the accelerated viral clearance and reduced morbidity levels observed during the resolution of the influenza infection in the PCN-treated mice was found to be dose dependent (Figure S1).

**Figure 4:**
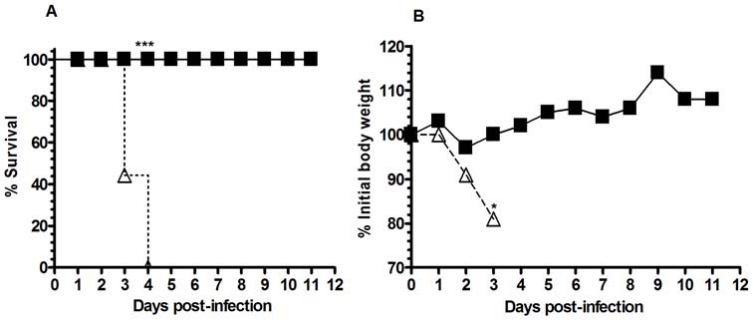
Survival of PCN and PBS-treated mice following challenge with SARS-coronavirus. A). All PCN-treated mice (▪) survived infection with SARS-coronavirus whereas all of the PBS-treated control mice (Δ) were dead by day 4 post-infection; ***, P<0.001. B) PCN-treated mice (▪) maintained their body weight following SARS-coronavirus infection whereas PBS-treated control mice (Δ) lost significant amounts of body weight shortly after infection.

To determine the extent to which this PCN treatment could provide a similar level of enhanced protection against other respiratory viruses we infected PCN- or PBS-treated mice with 1LD_100_ of a murine-adapted SARS-coronavirus (Figure 4). Notably, all PCN-treated mice survived the infection with minimal weight loss whereas all of the PBS-treated mice rapidly lost weight and died by day 4 post-infection (Figure 4A and 4B). Similarly, we found that PCN-treatment enhanced protection in mice infected either with an alternative influenza virus strain (X31 – H3N2) (Figure S2-A and S2-B) or infected with pneumovirus of mice (PVM) [13] (Figure S2-C and S2-D). Infection of mice with PVM results in a viral lung infection similar to that of RSV infections in humans [13]. To test whether this phenomenon is common to other PCN constructs, BALB/c mice were also treated with the human H-chain ferritin (HFn) protein cage and challenged with influenza virus. As a control, a group of mice were also treated with bovine serum albumin (BSA). Although the reduction in viral burdens were significant for mice treated with the HFN and for those treated with our PCN, this reduction in viral burden was 100 fold greater in the PCN treated mice (Figure S3-A). Viral burdens were not significantly different between the BSA-treated and PBS-treated mice. Neither HFn nor BSA treatments protected mice from weight loss following infection (Figure S3-B). Thus, it is evident that the overall effect of this PCN enhancement of immunity is not a generic feature of all PCN architectures and that the small heat-shock PCN appears uniquely well suited to facilitate the enhancement of protection against a broad spectrum of respiratory viruses.

**Figure 5:**
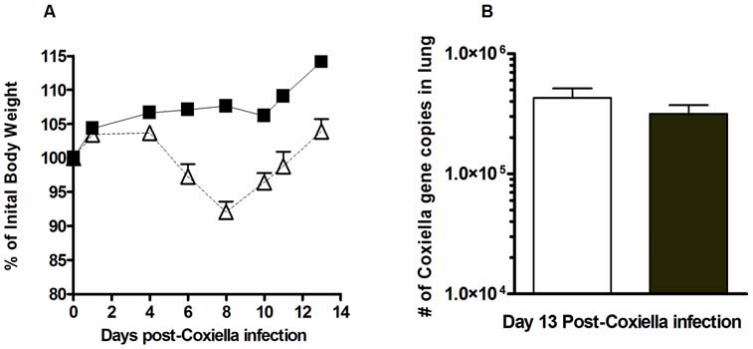
Resolution of a *Coxiella burnetii* infection in PCN-treated mice. A) PCN-treated mice (▪) gained body weight following *C. burnetii* infection whereas PBS-treated control mice (Δ) initially lost weight but were able to recover by 2 weeks post-infection. B) No difference in the rate of clearance of the *C. burnetii* infection from the lungs of the PCN-treated (shaded bar) and PBS-treated (clear bar) mice was detected.

The formation of iBALT structures in the PCN-treated mice (Figure 2A) and the increased cellular content recovered in the BALF of PCN-treated mice prior to their infection (Table 1) were indicative of the ability of the PCN to act as a pulmonary immunostimulatory agent. In spite of this PCN-driven immunostimulation, we did not observe any histological damage to the alveolar architecture of the lung following the PCN-treatment regimen. The lack of any lung damage associated with the PCN treatment was further supported by the absence of any increase in the levels of serum albumin (Figure 3C) and lactate dehydrogenase (Figure 3D) recovered in the BALF of these mice at this time and the absence of any weight loss during the PCN treatment regimen (data not shown). However, since it has already been demonstrated that novel small agents can act in an immunostimulatory role in the lung to enhance protective innate immune responses [14] against bacterial pathogens and that we could detect immunogenic changes in the lung following the PCN treatment regimen, we examined the potential of our PCN treatment regimen to enhance protection against a range of pulmonary bacterial pathogens. Three days after the conclusion of the 5x PCN treatment regimen, mice received a pulmonary challenge of 10^3^ colony-forming units of the phase 1 variant of *Coxiella burnetii*. Although the PCN-treated mice did not experience the level of weight loss observed in the PBS-control treated mice in the days following infection (Figure 5A), no difference in the rate of clearance of the *C. burnetii* infection was observed between the PBS-control and PCN-treatment groups (Figure 5B). We also tested this PCN treatment strategy in a similar fashion using *Francisella tularemia* and *Yersinia pestis* infection models, but again found no enhanced protection associated with the PCN treatment (data not shown). Thus the pulmonary immune environment elicited by this PCN treatment regimen seems to be uniquely suited to enhancing the resolution of subsequent primary viral infections but not subsequent bacterial infections of the lung.

**Table 1:** Total and differential cell counts recovered in BALF following 5x PCN-treatment regimen prior to infection.

Treatment	Total cells (×10^5^)	% Macrophages	% Lymphocytes	% Neutrophils
PBS treated	2.48±0.94	100	0	0
PCN treated	13.92±5.01**	72.8±4.2***	17.4±4.8***	9.6±4.0***

Three days after the 5x PCN treatment regimen, mice were sacrificed and lung lavages were carried out. Total and differential cell counts were determined. n = 5 mice per group; **, P = 0.001; ***, P<0.001.

Since the PCN was expressed and purified from an *E. coli* heterologous expression system, it is most likely to contain residual contaminating LPS. To investigate a potential adjuvant-like role for this LPS in the PCN-enhanced protection, we treated C3H/HeJ mice, which have decreased responsiveness to LPS [15], with PCN or PBS and then challenged them with PVM (Figure S2-C and D) or with influenza virus (Figure S4-A, B, and C). C3H/HeJ mice treated 3x or 5x with the PCN exhibited significantly reduced influenza viral burdens (Figure S4-A), less morbidity following influenza virus (Figure S4-B and S4-C) or PVM (Figure S2-C) infection, and significantly enhanced survival rates following PVM infection (Figure S2-D) relative to the PBS-treated controls. Furthermore, dramatically reducing the LPS in the PCN preparations (1.28 ng/dose) did not significantly alter the accelerated rate of viral clearance in mice that had been treated with this preparation (Figure S4-D). Finally, treatment with the HFn protein cage, which was also produced in *E. coli* and presumably with similar levels of LPS contamination, did not enhance protection against the influenza infection as well as treatment with the PCN preparation did (Figure S3). Taken together, these data strongly suggest that the presence of LPS is not a major contributor to the enhanced protection and that the small heat-shock PCNs are intrinsically immunomodulatory.

### T and B cell roles in enhanced protection

**Figure 6:**
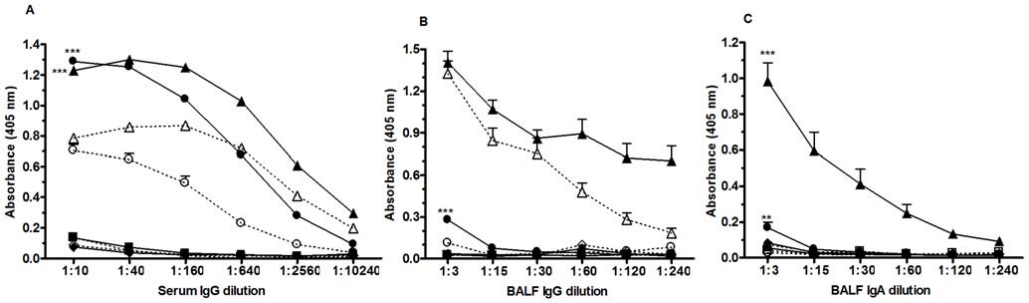
Kinetics of PR8 influenza-specific antibody response following infection in mice treated with PCN (filled symbols) or PBS (clear symbols) prior to infection. PR8 influenza-specific antibody levels were measured on day 0 (square symbol), day 5 (diamond symbols), day 7 (circle symbols) and day 9 (triangle symbols) post-infection. A) PR8 influenza-specific serum IgG levels appear sooner and are significantly enhanced in PCN-treated mice relative to the PBS-treated control mice; ***, P<0.0001. B and C) PR8 influenza-specific IgG and IgA levels appear sooner and are more intense in the BALF of PCN-treated mice following infection; **, P = 0.0002; ***,P<0.0001.

**Figure 7:**
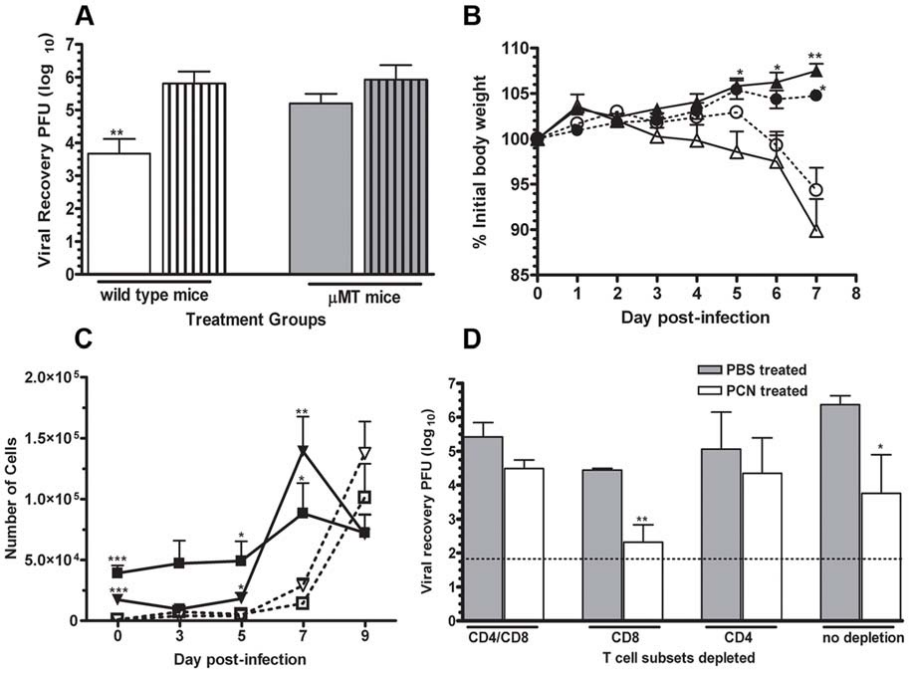
PCN-enhanced resolution of a PR8 influenza virus infection is antibody and CD4+ T cell dependent. A). Viral burden in lungs of PCN-treated (clear bars) wild type mice were significantly less than in the PBS-treated (clear bar with lines) wild type mice at day 7 post-infection whereas no differences in viral burdens in the lungs of the PCN-treated (shaded bars) and PBS-treated (shaded bars with lines) μMT mice were detected at this time; **, P<0.01 relative to PBS–treated control mice; limit of assay detection is 1.82 log_10_. B). Equivalent levels of body weight loss were detected in the PBS-treated wild type (Δ) and μMT (○) whereas PCN-treated wild type (▴) and μMT (•) both gained equivalent amounts of body weight during the week following influenza infection; *, P<0.05; **, P<0.001 relative to their respective PBS-treated control. C). Recruitment of CD8 (triangle symbols) and CD4 (square symbols) T cells occurs sooner in PCN-treated mice (filled symbols) relative to the PBS-treated (clear symbols) control mice following influenza virus infection; *, P<0.05; **, P<0.01; ***, P<0.001. D). Viral recovery in PBS-treated (shaded bars) and PCN-treated (clear bars) wild type mice depleted of CD8 and/or CD4 T cells at 48 hours before and 4 days after influenza infection; *, P<0.05; **, P<0.01 relative to PBS-treated control; dotted line at 1.82 log_10_ is limit of assay detection.

The appearance of PR8 influenza-specific serum IgG, BALF IgG, and BALF IgA responses was accelerated and significantly greater in the PCN-treated mice following influenza virus infection (Figure 6). This lead to speculation that the accelerated influenza-specific antibody response was a critical component of the underlying protective mechanism in the accelerated resolution of infection in the PCN-treated mice. To determine the necessity of the humoral immune response for this PCN-enhanced protection, B cell-deficient uMT mice were treated with either PCN or PBS prior to infection with influenza virus. We observed accelerated viral clearance in the PCN-treated wild type mice, but not in the PCN-treated uMT mice (Figure 7A), demonstrating the requirement of B cells for the enhanced clearance. However, that the PCN-treated uMT and wild type mice actually gained weight after infection whereas the PBS-treated uMT and wild type mice lost weight (Figure 7B) indicated that the protective effect of the PCN treatment against body weight loss is not wholly dependent on the presence of B cells. Thus, the PCN-induced mechanisms responsible for accelerated viral clearance may be distinct from those responsible for decreased weight loss.

We also observed that CD4 and CD8 T cells accumulated more rapidly in the lungs of PCN-treated mice following infection (Figure 7C) than in the lungs of PBS-treated mice. To determine whether CD4 or CD8 T cells played a role in PCN-mediated protection against influenza virus we depleted T cell subsets prior to and after infection. The accelerated viral clearance was still observed in PCN-treated mice that were depleted of CD8 T cells but not in those PCN-treated mice that were depleted of CD4 T cells or both CD4 and CD8 T cells (Figure 7D). Thus, CD8 T cells do not play a significant role in the enhanced protection against influenza infection in the PCN-treated mice, whereas, both B cells and CD4 T cells are critical for full protection.

### Role of iBALT in PCN-enhanced protection

**Figure 8:**
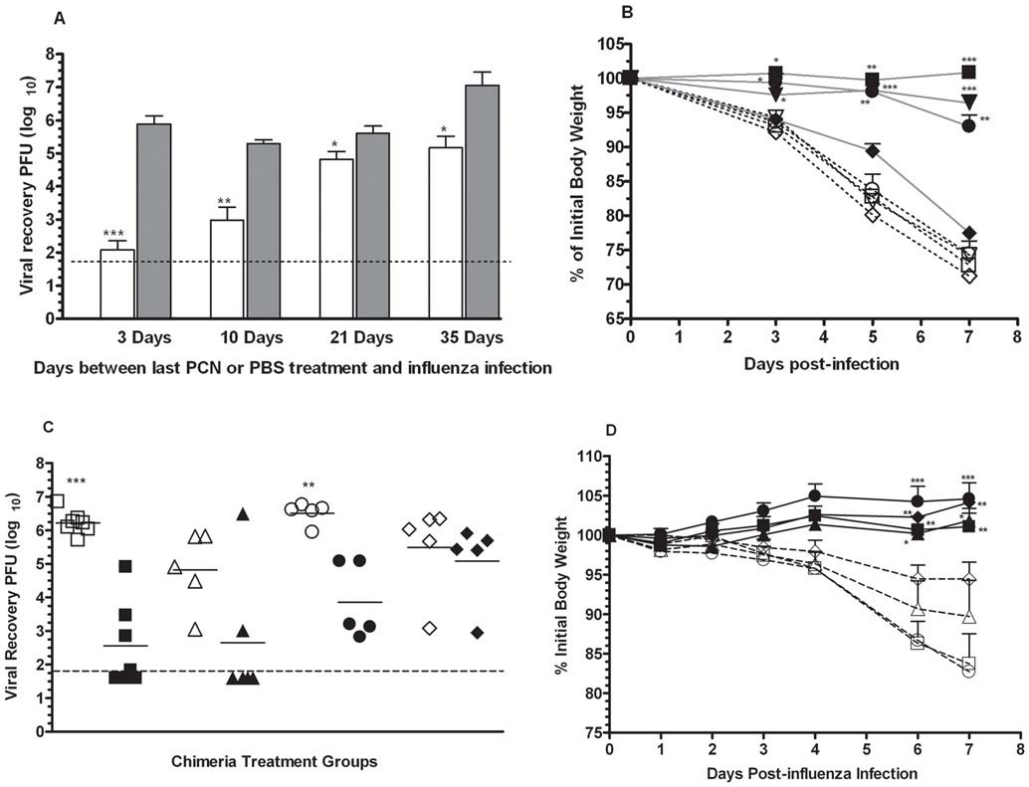
Effect of PCN treatment endures and elicits iBALT structures that can exclusively resolve PR8 influenza infections. A). Mice that had been treated with PCN (clear bars) or PBS (shaded bars) were rested for a designated period of time before being challenged with influenza virus. Assessment of viral clearance was carried out by plaque assay; *, P<0.05; **, P<0.001; ***, P<0.0001; dotted line is limit of assay detection. B). Weight changes in PCN-treated mice (closed symbols) and the PBS-treated control mice (open symbols) were monitored during the week following influenza virus challenge. Influenza virus challenge was given at 3 days (square symbols), 10 days (triangle symbols), 21 days (circle symbols) and 35 days (diamond symbols) after the conclusion of the PCN treatment regimen; *, P<0.05; **, P<0.005; ***, P<0.0005 relative to corresponding PBS-treated control. C). Splenectomized-irradiated mice were reconstituted with BMC in order to make chimeric mice in which responding iBALT structures could be isolated from all other conventional secondary lymphoid tissues. Wild type chimeric mice – wild type mice that received wild type BMC (square symbols), WT/LTα chimeric mice – wild type mice that received LTα KO BMC (triangle symbols), SLP chimeric mice - LTα KO mice that received wild type BMC (circle symbols) and LTα KO chimeric mice - LTα KO mice that received LTα KO BMC (diamond symbols). Following engraftment of the transferred BMC, groups of chimeric mice were treated 5x with PCN (solid symbols) or with PBS (open symbols) and then challenged with influenza virus. Viral burdens were determined 7 days after infection. **, P = 0.001; ***, P<0.0001; dotted line is limit of assay detection. D). Changes in body weight relative to that at the time of viral challenge were monitored daily in the PCN and PBS-treated chimeric mice; symbol designations as for Figure 8C; *, P<0.05; **, P<0.005; ***, P<0.0001 relative to corresponding PBS control.

Previous studies have indicated that the existence of iBALT in the lungs is transient [6]. To determine the duration of enhanced protection induced by the PCN treatment, PBS- and PCN-treated mice were challenged with influenza virus 3 to 35 days after completion of the PCN treatment. Although the protective effects of the PCN treatment waned as the interval between administration of the last PCN dose and the influenza infection increased, viral burdens in the PCN-treated mice infected 35 days after the last treatment were still significantly reduced relative to the PBS-treated control mice (Figure 8A). However, protection against weight loss was not as enduring (Figure 8B), further suggesting that the PCN-induced mechanisms responsible for accelerated viral clearance may be distinct from those responsible for decreased weight loss.

In order to determine whether PCN-induced iBALT structures could exclusively provide this enhanced protection, we generated chimeric mice by reconstitution of splenectomized, irradiated C57BL/6 wild type and lymphotoxin-α (LTα) deficient mice with wild type or lymphotoxin-α knockout (LTα-KO) bone marrow cells (BMC). Using these chimeric mice we were able to effectively isolate iBALT generated immunity from that generated by conventional secondary lymphatic tissues. The following 4 types of bone marrow chimeric mice were made; chimeras that lacked secondary lymph nodes (2^0^LN) and Peyers patches but could still form organized iBALT (LTα deficient mice that received wild type BMC - SLP chimeric mice); chimeras that lacked 2^0^LN and Peyers patches and were unable to form organized iBALT due to their lack of LT-α (LTα deficient mice that received LTα deficient BMC - LTα-KO chimeric mice); chimeras that retained 2^0^LN and Peyers patches but were likewise unable to form organized iBALT due to their lack of LTα (wild type mice that received LTα deficient BMC - WT/LTα chimeric mice); and wild type control chimeras that retained 2^0^LN and Peyers patches and could form organized iBALT (wild type mice that received wild type BMC - wild type chimeric mice) [7]. Following engraftment of the transferred BMC, the chimeras were treated 5x with PCN or PBS and then challenged with influenza virus 3 days after the last treatment. Seven days after infection, the PCN-treated wild type chimeric mice (filled squares) and SLP chimeric mice (filled circles) had significantly less virus in their lungs relative to their respective PBS-treated cohorts (open squares and open circles respectively) (Figure 8C). The extent of viral clearance in the PCN-treated WT/LTα chimeric mice (filled triangles) and LTα-KO chimeric mice (filled diamonds), both of which do not make organized iBALT [7], was closer to or equivalent to their respective PBS-treated mice (open triangles and open diamonds respectively). However, there was no weight loss in any of the PCN-treated chimeric mice (Figure 8D). Together, these results indicate that organized secondary lymphoid tissues are not required for the PCN-induced enhancement of protection against influenza virus infections and that pre-existing iBALT can exclusively account for the enhanced protection against the viral infection and is most likely also associated with the protection against weight loss.

The association of iBALT structures with lung pathology in auto-immune diseases [5] and our observation that PCN treatment altered lung micro-architecture, elicited an influx of leukocytes and enhanced local pulmonary immunity through iBALT formation suggested that this PCN treatment regimen may leave the lung susceptible to the development of allergic airway hyperresponsiveness. To assess this possibility, mice that had been systemically sensitized to ovalbumin were treated 5x with PCN or PBS, then given 3 intranasal instillations of ovalbumin and subsequently challenged with methacholine. Interestingly, airway hyperresponsiveness (Penh values) was attenuated in PCN-treated mice (Figure S5-A) and eosinophil influx into the lungs was reduced (Figure S5-B). These results indicated that the PCN-induced alterations to the lung did not render the lung susceptible to or exacerbate allergic airway hyperresponsiveness.

## Discussion

Our results clearly indicate that pulmonary instillation of PCN can dramatically enhance subsequent host immune responses to primary viral infections of the lung as well as curtailing the extent of pulmonary damage that these responses can cause. The absence of damage indicators recovered in the BALF (serum albumin and LDH) of the PCN-treated mice before infection, their reduced recoveries after infection and the lack of morbidity in these mice during the resolution of infection suggests that the PCN-treatment does not induce inflammatory damage in the lung. Even though the PCN treatment did alter pulmonary micro-architecture by inducing iBALT structures and was able to elicit an influx of leukocytes into the airways, these changes to the immune environment of the lung were of no apparent consequence to the well being of the mice. In fact, our results suggest that the PCN-treatment prior to infection is associated with an amelioration of the often excessive inflammatory responses that are seen during the resolution of pulmonary viral infections [16] as well as airway hyperresponsiveness and inflammation associated with asthma. Interestingly, the alterations to the immune environment of the lung in response to the PCN treatment had no effect upon the resolution of pulmonary bacterial infections.

The immunomodulatory effects elicited by the PCN-treatment are dependent on B cells and CD4 T cells, but not on either CD8 T cells or conventional secondary lymphoid tissues such as the spleen, lymph nodes and Peyer's patches. Our results strongly suggest that these immunomodulatory effects are mediated to a significant degree by the asymptomatically induction of iBALT in the lungs of recipient mice. This is consistent with previous results showing that pulmonary immune responses in the presence of iBALT are more efficient and less damaging to the host [7]. Because iBALT can serve as a functional lymphoid tissue at the site of infection, it is likely to acquire antigen more quickly and to respond to inflammatory signals in the lung more rapidly, thereby facilitating the acceleration of local adaptive immune responses. Given that antibodies are extraordinarily powerful at neutralizing virus, preventing further infection, clearing antigen and even dampening inflammation via FcR ligation, the accelerated IgG and IgA responses in the lung most likely contribute, at least partially, to the enhanced viral clearance in PCN-treated mice.

Adaptive immunity is probably not the only component of the immune system affected by PCN-treatment, since protection against a SARS-coronavirus infection was already apparent by 3 days after infection. This is well before an adaptive primary immune response expands to the point of effectiveness. Thus, it is likely that innate mechanisms are also modulated by this PCN treatment. How PCN treatment may enhance innate anti-viral mechanisms is currently unknown but our data suggests that it likely involves B cell and/or CD4 T cell-dependent mechanisms.

We believe that induction of iBALT in response to treatment with PCN represents a promising strategy for pulmonary immunoprophylaxis. Such a prophylactic strategy could enhance primary immune responses to a spectrum of respiratory viruses, making these responses not only more efficient at clearing the virus, but also less damaging to the lung in the process. The ability to non-specifically enhance immune protection against a diversity of respiratory viruses, most notably in the absence of significant pulmonary inflammation, underscores the potential effectiveness of this strategy against infections by antigenic variants of currently circulating viruses (H1N1 swine influenza pandemic, 2009), by newly emerging viruses (SARS epidemic, 2003) and by the numerous common respiratory viruses to which specific vaccines have not yet been successfully developed (RSV vaccines).

## Materials and Methods

### Mice and viruses

Male and female wild type C57BL/6 (CD45.2), BALB/c, and μMT (B10.129S2(B6)-Igh-6^tmlCgn^) mice were bred at Montana State University, Bozeman, MT. C3H/HeJ (Tlr4^Lps-d^) male and female mice were purchased from Jackson Laboratories (Bar Harbor, ME, USA). B6.SJL-Ptprc^a^, Pep3^b^/BoyAiTac mice expressing the congenic maker (CD45.1) were purchased from Taconic Laboratories (Hudson, NY, USA). Bone-marrow chimeric mice were generated from splenectomized-irradiated recipient mice as described [7]. The influenza virus strains A/PR8/8/34 (PR8; H1N1) and A/HKx31 (X31; H3N2) were obtained from the Trudeau Institute, Saranac Lake, NY, USA. The pneumovirus of mice (PVM) was a gift from Dr. Helene Rosenberg (NIAID, NIH, Bethesda, MD, USA). Work with the murine adapted SARS-CoV was carried out by Dr. Dale Barnard (Utah State University, Logan, UT, USA). All studies described here were approved by the IACUC board at Montana State University or Utah State University prior to their performance.

### Generation of bone marrow chimeric mice

Chimeric mice were generated by bone marrow reconstitution of irradiated C57BL/6 or lymphotoxin-α knockout (LT-α KO) mice that had been splenectomized 3 to 4 weeks earlier. Splenectomized recipient mice were gamma irradiated with 2 doses of 475 rads administered 4 h apart. The recipient mice were then immediately reconstituted by intravenous tail injection of 2×10^7^ congenic CD45.1^+^ or CD45.2^+^ donor bone marrow cells (BMC). The animals then rested for 5 weeks to allow for the establishment of the engrafted bone marrow cells. The discrepancy in CD45.1 and CD45.2 markers between donor and recipient mice was used to monitor the engraftment of the donor BMC whenever possible. The following chimeric mice were made; wild type CD45.1^+^ BMC into wild type CD45.2^+^ mice, wild type CD45.1^+^ BMC into LT-α KO CD45.2^+^ mice, LT-α KO CD45.2^+^ BMC into wild type CD45.1^+^ mice and LT-α KO CD45.2^+^ BMC into LT-α KO CD45.2^+^ mice.

### PCN Production

The genomic DNA of *Methanococcus jannaschii* was obtained from ATCC (43607). The cloning, characterization and purification of the small heat-shock protein (sHsp 16.5) from this archaeon was carried out in an *E. coli* expression vector as described previously [17]. The purified PCN was then stored at −80°C until used.

### Inoculation procedures and tissue recovery

Administration of the PCN and viral infections were carried out while mice were under a light anesthesia using 5% isoflurane inhalation. The PCN inoculations contained 100 µg per dose, the PR8 influenza inoculum contained 1500 PFU, the X31 inoculum contained 4×10^6^ PFU. The PVM inoculation was a 1∶200 dilution of our viral stock. The SARS-CoV infections were carried out using a 1LD_100_ of the viral stock. *Coxiella burnetii* infections were carried out using 10^3^ genomic equivalents of the phase 1 variant. Daily body weights of the mice were monitored following infection.

At designated times following viral infection, mice were sacrificed by intraperitoneal administration of a lethal dose of sodium pentobarbital (90 mg/kg) followed by exsanguination when the mice no longer exhibited a pedal reflex. Lungs were then harvested, snap-frozen in liquid nitrogen and then stored at −80°C for future use in viral plaque assays or for recovery of bacterial genomic copies. Lungs used for immuno-histological staining were instilled with O.C.T. Tissue Tek (Sakura Finetek USA Inc., Torrance CA, USA), snap frozen on liquid nitrogen and stored at −80°C for future use. Lung sections were cut and stained with FITC-conjugated anti-mouse B220, PE-conjugated anti-mouse CD4 or PE-conjugated anti-mouse CD21 (BD Biosciences San Diego CA, USA).

### Recovery of broncho-alveolar lavage fluid

Broncho-alveolar lavage fluids (BALF) were obtained by washing the lungs with 2.0 ml of 3 mM EDTA in HBSS in two aliquots of 1 ml. Cells recovered in the BALF were counted and then spun out of the recovered BALF. The recovered fluid was then stored at −80°C for future use in determining levels of serum albumin, lactate dehydrogenase, and influenza-specific antibody.

### Serum albumin and lactate dehydrogenase content of BALF

The serum albumin content of the BALF was determined by use of an albumin colorimetric assay (Sigma-Aldrich, St. Louis MO, USA). Color absorbency was read at 630 nm and reported in mg/ml of BALF. The level of LDH detected in the BALF was determined by colorimetric assay (CytoTox 96, Promega, Madison WI, USA). The assay absorbance was read at 490 nm and reported as U/ml in the BALF. Each of the samples was tested in duplicate.

### Depletion of T cell subsets

Depletion of CD4 and CD8 T cell subsets was accomplished by intraperitoneal injection of specific monoclonal antibodies (MAb). CD4 T cells were depleted by injection of 300 ug of the anti-mouse CD4 MAb, GK1.5. CD8 T cells were depleted in a similar manner using the anti-mouse CD8 MAb, TIB 210. Injections of these monoclonal antibodies were given 24 hours prior to infection and 4 days after infection. Using this method, >95% depletion of the selected T cell subsets endured for the duration of the experiment. Both of these MAbs were made in house using in vitro tissue culture.

### Determination of influenza-specific antibody levels

Determination of the PR8-specific antibody levels in the serum and BALF was carried out as described previously [18]. Briefly, 100 ul aliquots of a 1/100 dilution of serum or 100 ul aliquots of the BALF were added to 96-well high binding polystyrene ELISA plates that had already been coated with our PR8 influenza viral membrane preparation and subsequently blocked with 5% nonfat skim milk. The plates were allowed to incubate for 2 h at 37°C. Following 3 washes with PBS-Tween, 100 ul of a secondary alkaline phosphatase-conjugated anti-mouse isotype-specific antibody (Southern Biotechnology Associates, Birmingham AB) was added. The plates were then allowed to incubate for 2 h at 37°C. The presence of bound Ab was detected by the addition of *p*-nitrophenyl phosphate in diethanolamine buffer and visualized at 405 nm.

### Titration of viral recovery from lungs

Our previously described plaque assay procedure was used in the experiments reported here [19]. Briefly, serial 10-fold dilutions of the lung homogenates were used to inoculate confluent monolayers of Madin-Darby canine kidney cells. Following the inoculation procedure, the assay was then allowed to incubate at 35°C for 3–4 days prior to being fixed in 20% acetic acid and stained with 0.2% crystal violet to reveal the presence of viral plaques.

### Airway hyperresponsiveness

BALB/c mice were sensitized to ovalbumin (ova) by intraperitoneal injection of 100 ug ova/4 mg alum adjuvant and then rested 10 days before receiving 5 intranasal 100 ug PCN instillations over a 2 week period. Five days after the last intranasal PCN instillation, mice were given an intranasal challenge of 20 ug ova in PBS for 3 consecutive days. On the following day, Penh value estimates of airway hyperresponsiveness to methacholine dose response challenges were monitored using a Buxco whole body plethysmograph.

## Supporting Information

Figure S1 PCN-induced enhancement of the resolution of a subsequent PR8 influenza virus infection is reduced as size of the PCN dose diminishes. Mice were treated 5x with designated doses of PCN or with PBS (0 ug PCN) via intranasal instillation over 12 days. Three days after the last treatment, mice were infected with 1500 PFU of the H1N1-PR8 strain of influenza virus. A) Viral recoveries were determined 7 days after infection by plaque assay; dotted line is limit of assay detection; ***, P<0.0001, **, P<0.005, *, P<0.05 relative to PBS group (0 ug PCN). B) Daily percentage weight changes from the initial body weights at the time of infection were determined; ***, P<0.001 vs 0 ug PCN; **, P<0.01 vs 5 ug PCN; *, P<0.05 vs 5 ug PCN; n = 6 mice per group.
(8.17 MB TIF)

Figure S2 Resistance to infection by X31 influenza virus or pneumovirus of mice is enhanced in mice treated with PCN prior to viral challenge. A) Recovery of X31 influenza virus by day 7 post-infection is significantly accelerated relative to PBS-control treated mice; ***, P = 0.0002; dotted line is limit of assay detection. B) Weight loss in PCN- (▪) and PBS- (□) treated mice during resolution of an X31-influenza infection; *, P = 0.04; ***, P≤0.0001. C) C3H/HeJ mice treated with PCN (▪) prior to infection with PVM retained or gained body weight following infection whereas PBS-treated control mice (□) lost body weight during resolution of the infection; *, P<0.05; **, P<0.01; ***, P≤0.0005. D) Survival of PVM infection is significantly enhanced in C3H/HeJ mice that were treated with PCN (▪) prior to infection.
(9.40 MB TIF)

Figure S3 PCN treatment uniquely enhances resolution of a PR8 influenza viral infection. A). Treatment of mice with bovine serum albumin or a human H-chain ferritin cage prior to viral challenge did not elicit an equivalent enhancement in the resolution of a subsequent influenza infection as seen for the PCN-treated mice; *, P<0.05; **, P<0.005 relative to PBS-treated mice; dotted line is limit of detection in the assay. B) Mice treated with PCN prior to viral challenge did not lose body weight during resolution of infection whereas mice receiving alternative treatments do lose body weight; **, P<0.01.
(2.13 MB TIF)

Figure S4 Resolution of a PR8 influenza virus infection in C3H/HeJ mice treated with PCN prior to challenge. A) Treatment of C3H/HeJ mice 3x or 5x with PCN resulted in an accelerated rate of viral clearance relative to PBS-treated controls as determined by viral burdens in the lungs of mice at day 7 post-infection; *, P<0.05; **, P<0.005; dotted line is limit of assay detection. B and C) Loss of body weight during resolution of infection was significantly less in C3H/HeJ mice that received PCN treatment prior to infection; **, P<0.01; *, P<0.05 relative to PBS treated control group. D). Viral recoveries at day 7 post-infection from lungs of mice treated 3x with PCN containing reduced endotoxin levels (1.28 ng/dose); **, P = 0.0014; dotted line is limit of assay detection.
(9.59 MB TIF)

Figure S5 Treatment with PCN ameliorates methacholine-induced airway hyperresponsiveness in ovalbumin sensitized mice. Mice were sensitized to ovalbumin by intraperitoneal injection followed 4 weeks later by 3 intranasal instillations of ovalbumin on 3 consecutive days. From 11 to 26 days after the intraperitoneal ovalbumin injection, mice were treated 5x with the PCN or PBS-control on every 4th day. On the day after the last intranasal ovalbumin instillation all of the mice were subjected to a methacholine challenge and measurement of their airway hyperresponsiveness was taken. A). Airway hyperresponsiveness (Penh values) in ovalbumin sensitized PCN-treated (solid squares), PBS-treated (open squares) mice and non-ovalbumin sensitized PBS-treated mice (open triangles) in response to methacholine challenge doses were recorded; *, P<0.05; Penh values for doses greater than 10 mg/ml methacholine were not taken for the PBS-treated ovalbumin mice due to severity of the airway hyperresponse reaction. B) Eosinophil influx into the airway was calculated from the differential cell counts of the recovered BALF; **, P<0.005.
(2.26 MB TIF)
