# Supplementary figures and images for: Inducible Bronchus-Associated Lymphoid Tissue Elicited by a Protein Cage Nanoparticle Enhances Protection in Mice against Diverse Respiratory Viruses
Source: PLoS One. 2009 Sep 23;4(9):e7142. doi: 10.1371/journal.pone.0007142 (PMC2743193; doi:10.1371/journal.pone.0007142)

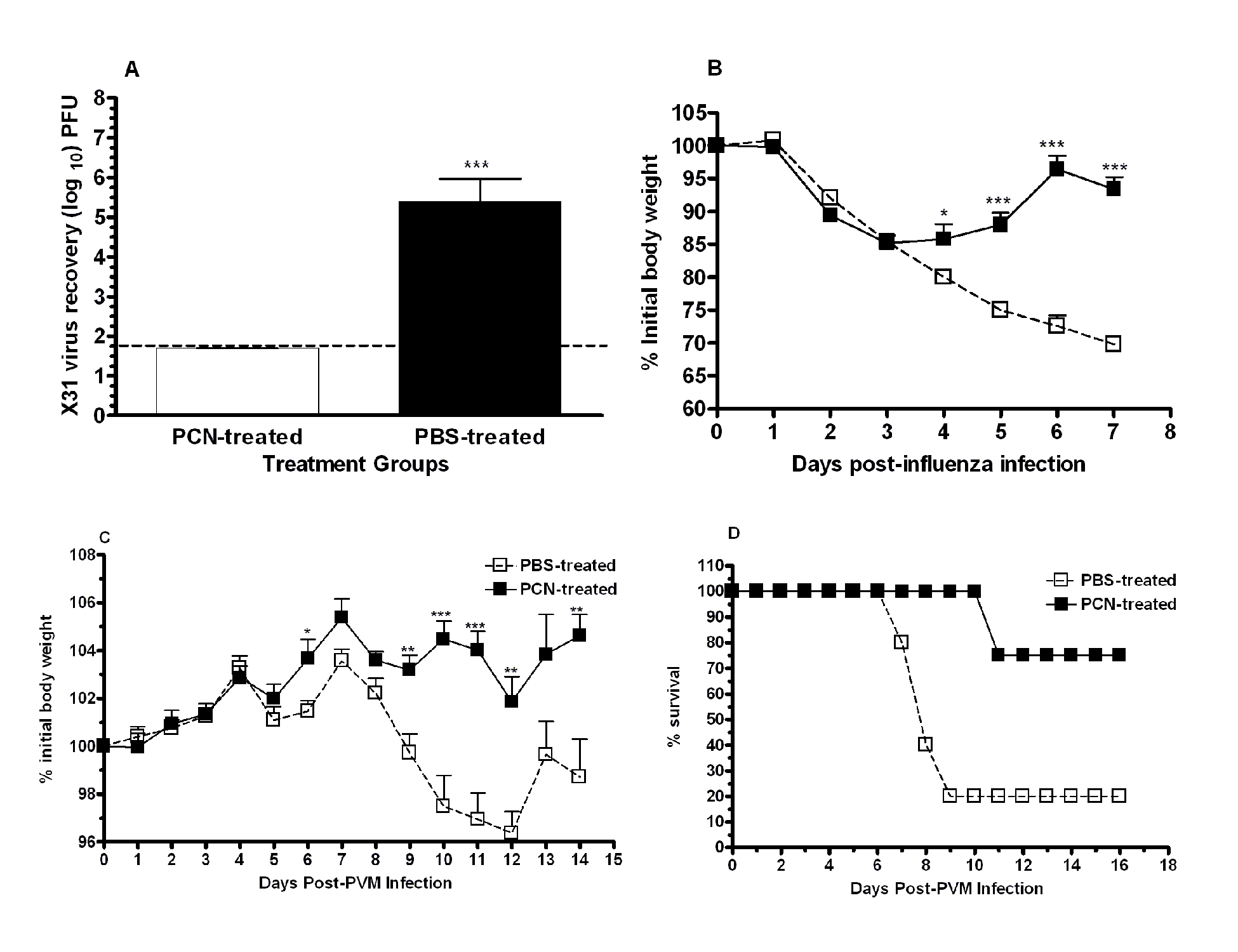

Supplement: Figure S2 — Resistance to infection by X31 influenza virus or pneumovirus of mice is enhanced in mice treated with PCN prior to viral challenge. A) Recovery of X31 influenza virus by day 7 post-infection is significantly accelerated relative to PBS-control treated mice; ***, P = 0.0002; dotted line is limit of assay detection. B) Weight loss in PCN- (▪) and PBS- (□) treated mice during resolution of an X31-influenza infection; *, P = 0.04; ***, P≤0.0001. C) C3H/HeJ mice treated with PCN (▪) prior to infection with PVM retained or gained body weight following infection whereas PBS-treated control mice (□) lost body weight during resolution of the infection; *, P<0.05; **, P<0.01; ***, P≤0.0005. D) Survival of PVM infection is significantly enhanced in C3H/HeJ mice that were treated with PCN (▪) prior to infection. (9.40 MB TIF) [file pone.0007142.s002.tif]

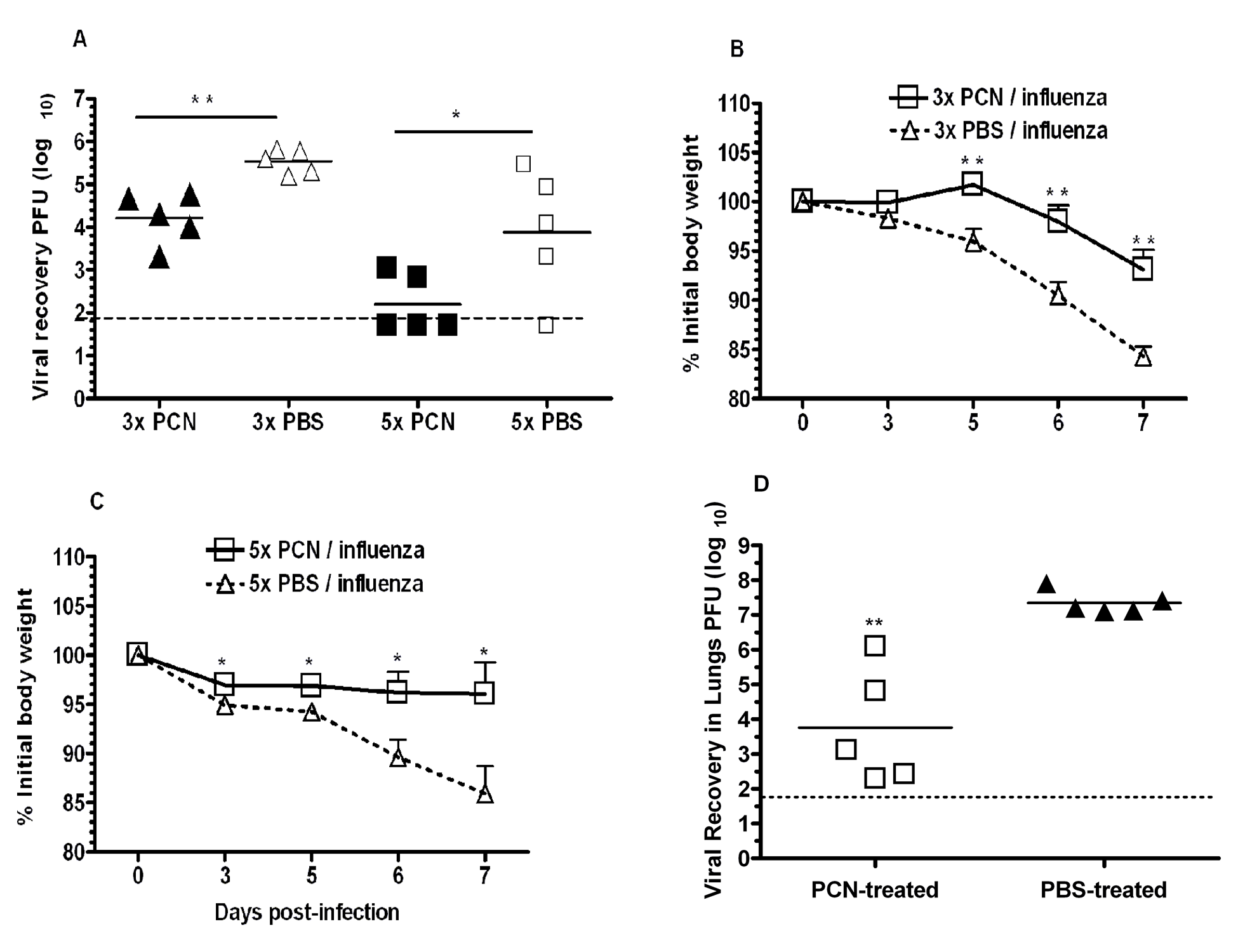

Supplement: Figure S4 — Resolution of a PR8 influenza virus infection in C3H/HeJ mice treated with PCN prior to challenge. A) Treatment of C3H/HeJ mice 3x or 5x with PCN resulted in an accelerated rate of viral clearance relative to PBS-treated controls as determined by viral burdens in the lungs of mice at day 7 post-infection; *, P<0.05; **, P<0.005; dotted line is limit of assay detection. B and C) Loss of body weight during resolution of infection was significantly less in C3H/HeJ mice that received PCN treatment prior to infection; **, P<0.01; *, P<0.05 relative to PBS treated control group. D). Viral recoveries at day 7 post-infection from lungs of mice treated 3x with PCN containing reduced endotoxin levels (1.28 ng/dose); **, P = 0.0014; dotted line is limit of assay detection. (9.59 MB TIF) [file pone.0007142.s004.tif]
